# Factors constraining the adoption of soil organic carbon enhancing technologies among small-scale farmers in Ethiopia

**DOI:** 10.1016/j.heliyon.2021.e08497

**Published:** 2021-11-27

**Authors:** Wilson M. Nguru, Charles KK. Gachene, Cecilia M. Onyango, Stanley K. Ng'ang'a, Evan H. Girvetz

**Affiliations:** a Department of Land Resource Management and Agricultural Technology, University of Nairobi, P.O. Box 29053-00625 Kangemi, Nairobi, Kenya; b Plant Science and Crop Protection Department, University of Nairobi, P.O. Box 29053-00625, Kangemi, Nairobi, Kenya; c International Centre for Tropical Agriculture (CIAT), Kampala 6247, Uganda; d International Center for Tropical Agriculture (CIAT), P.O Box 823-00621 Nairobi, Kenya

**Keywords:** Sustainable land management, Adoption, Soil organic carbon, Ethiopia, Small-scale farmers, Technologies

## Abstract

Declining soil fertility is one of the major causes of food insecurity and high levels of poverty, both of which tend to hamper economic development in sub-Saharan Africa (SSA). To improve soil fertility, the implementation of soil organic carbon (SOC) enhancement technologies has become crucial to slowing land degradation, through increasing SOC, which is the basis of soil fertility. Using data from 381 households from Azuga-Suba and Yesir watersheds in Ethiopia, this study explores the extent of the adoption of technologies that enhance SOC. Soil organic carbon enhancing technologies include the use of manure, fertilizer, and crop residue management. The Probit model was used to assess what constrains the adoption of these technologies. The results indicate that fertilizer is the most adopted technology having over 90% adoption in both watersheds. Manure at 28% and 56% adoption while crop residue management at 37% and 26% adoption in Azuga-Suba and Yesir respectively. Technology adoption is highly constrained by lack of education, access to extension services, and access to credit services. Institutions and local farmer groups influence these constraints through training, provision of information, offering incentives, and credit services. Large plots hinder the use of manure and fertilizer due to the bulky nature of manure and the high costs of fertilizers. Insecurity in land tenure limits the adoption of manure and residue management. Perception of soil erosion and soil fertility tends to constrain the adoption of SOC technologies, as farmers are afraid that all improvements through soil amendment will be diminished through soil erosion. At the same time, farmers do not perceive the importance of SOC enhancing technologies in plots that were fertile. These results imply that strengthening institutions that enhance farmers’ knowledge and provide credit as well as strengthening social protection schemes and farmer groups is crucial in promoting the adoption of these technologies.

## 1 Introduction

In Ethiopia, agriculture accounts for 46% of the GDP, with exports amounting to 84%, and employs more than 80% of the population (Belay, 2020; Werku, 2014). Soil degradation is the main cause of decreased agricultural production and results from anthropogenic factors (Gomiero, 2016). It leads to poverty, hunger, malnutrition, and even death. This results from a rapidly increasing population, without a counter mechanism to increase food production (Ramakrishna and Demeke, 2005). Ethiopia has an annual growth rate of approximately 2.6% in Ethiopia and the food production is not enough to feed this increasing population. This translates to farmers practicing continuous cropping on their farmlands and the expansion of the agricultural lands into marginal lands (FAO, 2013). Consequently, this leads to an increase in pressure on farmlands that results in the depletion of soil nutrients, thereby rendering the soil unproductive.

Reduced soil fertility is attributed to low soil organic carbon (SOC). Soil carbon is an essential component of healthy ecosystems, as well as a source of food, soil, water, and energy (Stockmann et al., 2015). It is the constituent of soil organic matter (SOM) that can be measured (Lal, 2015; FAO, 2017). Soil organic matter (SOM) is the organic component of soil, excluding undecayed plant and animal remains (Sikora and Stott, 2015). Approximately, between 2–10% of the soil's mass is made up of organic matter (Schjønning et al., 2018). Organic matter (OM) performs a vital role in agricultural soils by enhancing their chemical, biological and physical performance (Schmidt et al., 2011). Soil organic matter, which is the remains of OM after losses, is usually impacted by climate, soil type, and land use management (Angers et al., 1997). It enhances nutrient retention and turnover, supports the soil structure, enhances soil water retention, enhances sequestration of carbon dioxide (CO2), assists in the degradation of pollutants, and increases the resilience of the soil (FAO, 2005). It rises significantly whenever the proportion of inputs in terms of residues, is higher than the proportion of losses (Grains Research and Development Corporation (GRDC), 2013). Inputs are determined by plant residue production, but in some cases may result from the addition of amendments to the soils or animal by-products (FAO, 2005). Losses of SOM occur due to various reasons such as decomposition, mineralization, soil erosion, and the reduced activity of decaying microorganisms (Viscarra et al., 2014).

Therefore, SOC is principal to the increase in soil fertility and productivity and consequently reducing poverty and malnutrition. Increasing SOC can be done by adopting soil organic carbon-enhancing technologies (SOCETs) that enhance stubble retention onto the farmlands, covers the soil, and reduce loss of soil and OM through erosion (FAO, 2017). These technologies encourage carbon sequestration in the soil, leading to the creation of a carbon sink (Smith et al., 2016; Kern and Johnson, 1993). Dahal and Bajracharya (2010) document these technologies as minimum tillage and no-tillage farming with the application of mulch and planting cover crops such as teff, wheat, finger millet, and legumes. Others include integrated nutrient management (INM), applying inorganic and organic fertilizers in a balanced application, crop rotations of cereals with legume crops and agroforestry, and improved rangelands with controlled rates of animal stocking (Dahal and Bajracharya, 2010). It is imperative to note that, the rate of SOC sequestration with the adoption of these technologies is dependent on soil texture and structure which vary depending on the type of soil, temperature, rainfall, farming system, and soil management (Dahal and Bajracharya, 2010).

Despite the many benefits associated with the adoption of these SOCETs as well as the considerable efforts carried out by the government, national and international organizations aimed at encouraging farmers to invest in them, the extent of adoption remains low (Ngongo, 2016; Teklewold et al., 2013). In Ethiopia, for instance, numerous soil and water conservation technologies have been invested to curb land degradation but the adoption remains low. In Kenya, through Kenya Agricultural and Livestock Research Organization (KALRO) and agricultural university research centres, the government together with private development partners have facilitated and introduced various soil and water conservation technologies through state and private funded agricultural research activities but the spread and extent of adoption of these technologies remains very low. Ingold (2002) observed that the failure of small-scale farmers to accept, apply and adopt land management technologies aimed at increasing productivity in their farms has led to extremely low agricultural productivity. This has led to increased food insecurity, poverty, hunger, and malnutrition in sub-Saharan Africa (SSA). The extent of land management technologies adoption is influenced by an array of factors, which have been largely categorized into; social, economic, and institutional factors (Mwangi and Kariuki, 2015). The adoption of SOCETs by smallholder farmers has been challenged by a variety of environmental, social, economic, and political characteristics explicit to the setting within which they are being adopted (Bisaro et al., 2011; Cordingley et al., 2015). The identified economic factors include land size, cost of technology or its anticipated advantages compared to the cost of adoption, and the farmers' financial state derived from off-farm undertakings (Dessart et al., 2019). Social factors that influence the possibility of a farmer adopting a technology include; age, level of education, gender, and social groupings (Kinyangi, 2014). Institutional variables that determine uptake of SOCETs include access to information, government policies, and access to extension services (Kinyangi, 2014). Environmental factors include climate and topography where farmers are unable to control them but they can adapt their crop management to mitigate constraints associated with these factors (Mariano et al., 2012). There is thus a need to speed up the rate of adoption of land management technologies that enhance SOC to improve food security. This, therefore, calls for a requirement of knowledge and understanding of the factors that constrain the small-scale farmer's decisions to adopt these technologies.

This study sought to establish the extent of adoption of the SOCETs and the factors constraining the adoption of these technologies by smallholder farmers in Ethiopia in a bid to increase SOC and consequently soil fertility. The present study utilizes plot-level information, household socioeconomic characteristics, and external support factors as the explanatory variables. The specific objective of this study was to assess the extent of adoption and the factors that constrain the adoption of SOCETs. The extent of adoption was measured by the percentage of farmers that have adopted each SOCET. The SOCETs considered in this study include manure, fertilizer, and crop residue management which were the dependent variables. These practices were considered due to their availability and ease of adoption in addition to their immediate impact on improving SOC. This is in comparison with other practices such as agroforestry crop rotation, intercropping, and grass strips which take longer to enhance soil organic carbon and consequently soil fertility.

## 2 Materials and methods

### 2.1 Description of the study site

**Figure 1:**
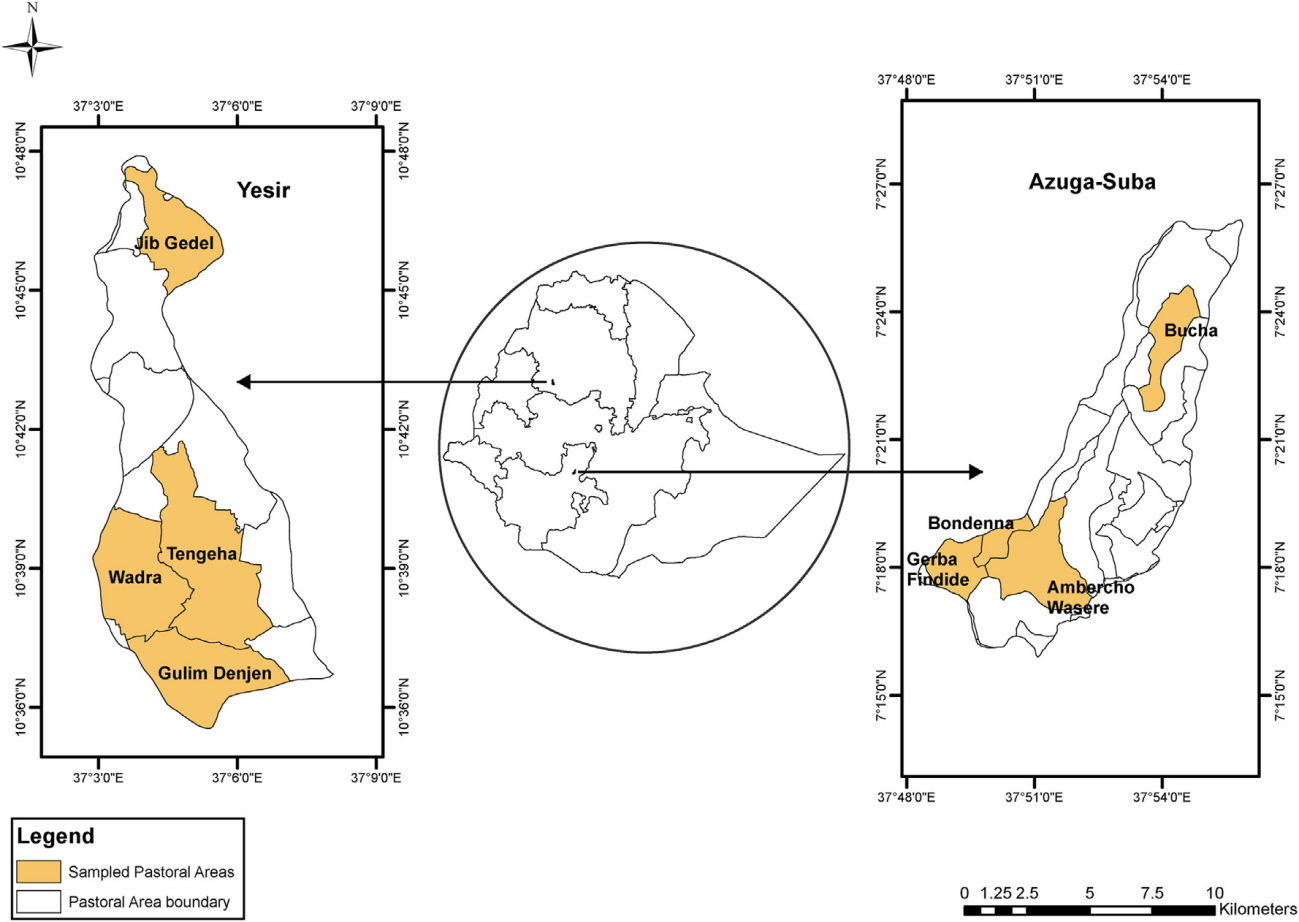
Map of the study areas.

This study was conducted in Yesir and Azuga-Suba watersheds in Ethiopia (Figure 1). Yesir watershed lies between longitudes 37°02′ and 37° 07′ East and latitudes 10° 35′ and 10° 48′ North and covers an area of about 116 km^2^ with a population density of approximately 158 persons per km^2^, while Azuga-Suba watershed lies between longitudes 37° 48′ and 37° 55′ East and latitudes 7° 15′ and 7° 26′ North and covers an area of about 89 km^2^ with a population density of approximately 502 persons per km^2^. The climate of Yesir watershed is characterized by a mean annual rainfall and mean annual temperature of 1817 millimeters (mm) and 19 degrees Celsius (°C) respectively (National Meteorological Agency, 2020). The dominant soils are Luvisols, Leptosols, Vertisols, and Nitisols (Hengl et al., 2017). Azuga suba watershed, on the other hand, receives a mean annual rainfall and mean annual temperature of 2043 mm and 20 °C respectively (National Meteorological Agency, 2020). The dominant soils are Vertisols and Luvisols with traces of Nitisols and Phaeozems to the south (Hengl et al., 2017).

Smallholder farmers in these watersheds practice a mixed system of crop and livestock in which crop and livestock mutually benefit from one another. The main crop management practices associated with these watersheds include the application of manure sourced from the livestock kept and crop rotation. The most common crop in Yesir and Azuga suba is teff (*Eragrostis tef (Zucc.) Trotter, 1918*), barley (*Hordeum vulgare Linnaeus, 1753*), wheat (*Triticum aestivum Linnaeus, 1753*)*,* and horse beans (*Vicia faba Linnaeus, 1753*). These crops are grown mostly for subsistence purposes. Other important crops include; maize (*Zea mays Linnaeus, 1753*), sorghum (*Sorghum bicolor (L.) Moench, 1794*), finger millet (*Eleusine coracana Linnaeus, 1759*), Enset (*Ensete ventricosum (Welw.) Cheesman, 1948*), pulses, and oil crops. Cattle (*Bos taurus Linnaeus, 1758*), goats (*Capra hircus Linnaeus, 1758*), sheep (*Ovis aries Linnaeus, 1758*), and poultry form the major types of livestock kept. In addition, donkeys (*Equus asinus Linnaeus, 1758*), horses (*Equus caballus Linnaeus, 1758*), and mules are also common. The study areas experience bimodal types of rainfall with the main season (***meher***) between June and September while the short rainy season (***belg***) is experienced between February and April (Mekonen et al., 2020).

### 2.2 Sample selection

The sampling frame comprised smallholder farmers in the Ethiopian watersheds of Yesir and Azuga-Suba. To obtain a household sample that is representative of various households and landscapes, both purposive selection (Tongco, 2007) and multi-stage random sampling (Raina, 2014) were adopted. The first stage involved the purposive selection of the two watersheds of Ethiopia (i.e., Yesir and Azuga-Suba). The multi-stage sampling procedure was then applied as follows; the first stage involved a random selection of pastoral areas (PAs) (also locally referred to as Kebeles) and which are the smallest administrative unit of Ethiopia. The selection of PAs involved the division of each watershed into three zones: upper, middle, and lower. The middle zone was divided into a lower and upper-middle zone. This was followed by the selection of one PA from the upper zone, two PAs in the middle zone (lower-middle and upper-middle), and one PA in the lower zone. This was done to acquire representative landscapes in the watersheds. In Yesir watershed, the selected PAs were Gulim, Jib Gedel, Tengeha, and Wadra while in Azuga-Suba watershed the selected PAs were Ambercho Wasere, Bondenna, Bucha, and Gerba Findide.

The target sample was determined using Eq. 1 and Eq. 2 (as discussed in Taherdoost (2017)) in order to arrive at about 320 households for both watersheds and 160 households for each.

(1) $$n_{0}=\frac{z^{2}pq}{e^{2}}$$

(2) $$n_{0}=\frac{1.96^{2}\left(0.5\times 0.5\right)}{0.055^{2}}=317\;\text{approximately}\;320$$

where $n_{0}\;$ is the sample size, $Z^{2}$ is standard normal deviate at the selected confidence level (which is 1.96 for commonly used 95% confidence interval), p is the estimated proportion of an attribute that is present in the population, q is 1−p and *e* is the desired level of precision.

A list of households in the two watersheds was drawn with the help of local administrators and extension officers, and sample households were randomly selected from this list. Only the households that owned a plot within the watersheds in the four selected PAs were included in the sampling and when the selected household was not available, the next available household on the list replaced it. In each watershed, additional households were interviewed to cater for data problems that could be associated with missing data or incompletely filled questionnaires. The ultimate sample size reached 219 observations in Yesir watershed and 162 in Azuga-Suba watershed. The total sample size, therefore, was 381 households. Internal farm divisions by individual farmers to plots led to a total of 2,602 plots in both watersheds with Azuga-Suba having 1,150 plots with an average plot size of 0.66 acres and Yesir having 1,452 plots with an average plot size of 0.58 acres.

During data collection, the targeted respondents were the household heads but in case they were absent, a member of the household with a good knowledge was identified to stand in as the respondent.

### 2.3 Data and data sources

Using a structured questionnaire, quantitative and qualitative data were collected. The information that was gathered during the survey included; households' demography characteristics, household wealth indicators, livestock holding, plot-level data, agricultural technologies and activities, inputs use, marketing activities, households' accessibility to markets, households' accessibility to credit services and households’ access to extension and training.

### 2.4 Dependent and explanatory variables

The collected data were summarized into dependent and explanatory variables (Table 1). Household size was measured as the number of household members who lived and ate in the same household. Distance to the plot and the market was measured in terms of the time (in minutes) taken to walk in minutes to the plot or the market respectively. Residue management was measured by assessing the crop residues on their plots after harvesting. Annual precipitation and slope were obtained from WorldClim (Fick and Hijmans, 2017) and USGS earth explorer (USGS, 2020) respectively and extracted for all households based on their location.

**Table 1:** Key variables for households in Ethiopia.

Dependent variables (dummy: 1 = Yes; 0 = No)	Mean ± S.D.	Min	Max
*Technology adoption*			
Manure	0.43 ± 0.50	0	1
Fertilizer use	0.95 ± 0.23	0	1
Residue management	0.31 ± 0.46	0	1
***Explanatory variables (plot-level)***			
Slope (*degrees*)	3.73 ± 3.19	0.11	20.8
Tenure security of the plot (*dummy, 1 = Yes; 0 = No*)	0.84 ± 0.37	0	1
Soil erosion perception (*dummy, 1 = Yes; 0 = No*)	0.24 ± 0.43	0	1
Plot size (*acres*)	0.61 ± 0.57	.0025	6.18
Distance to plot (*walking minutes*)	17.04 ± 23.03	0	210
Plot fertility perception (*dummy, 1 = Yes; 0 = No*)	0.92 ± 0.28	0	1
*Socioeconomic variables*			
Education level of household head (grade/level)	2.61 ± 1.04	1	5
Livestock ownership (*number*)	0.98 ± 0.14	0	1
Household years in farming	25.95 ± 11.72	2	60
Household size (*number*)	6.60 ± 2.13	1	13
Distance to urban market (minutes)	88.62 ± 54.17	7	240
*Institutional characteristics*			
Credit access (*dummy, 1 = Yes; 0 = No*)	0.28 ± 0.45	0	1
Access to extension (*dummy, 1 = Yes; 0 = No*)	0.79 ± 0.41	0	1
Group membership (*dummy, 1 = Yes; 0 = No*)	0.57 ± 0.50	0	1
*Climatic characteristics*			
Annual precipitation (mm)	1442 ± 227.82	1105	1828

#### 2.4.1 The dependent variables

Three dependent variables are considered in Table 1. They include manure, fertilizer use and residue management. Manure involves the application of animal waste on the crop plot. Fertilizer use involves the addition of chemical elements into the soil to supplement the missing soil nutrients in a crop plot. Residue management involves leaving crop residues from the previous season after the harvest to improve the soil's physical and chemical properties (McSorley and Gallaher, 1994).

#### 2.4.2 The explanatory variables

##### 2.4.2.1 Variables describing plot-level characteristics

Plot specific variables include plot slope, tenure security, soil erosion perception, size and soil fertility perception of the plot and distance to the plot.

##### 2.4.2.2 Socioeconomic variables

Socioeconomic variables that were considered in this study include distance to the urban market, education level of household head, livestock ownership, household years in farming and the size of the household.

##### 2.4.2.3 Institutional characteristics

Institutional characteristics that were taken into account in this study were farmers’ access to credit, access to extension and membership of the household in farmer groups and associations.

##### 2.4.2.4 Climatic characteristics

Annual precipitation/rainfall is the only climatic characteristic considered study. Precipitation affects soil erosion and the growth of vegetation. In highly sloping areas farmers tend to adopt various SOCETs to control water flow and consequently soil erosion.

### 2.5 Data analysis

Data were analyzed using the Stata program version 14.1. The Probit model established the relationships between each SOCETs and the explanatory variables. The Probit model, therefore, enabled us to analyze the variables influencing the likelihood of adoption, which could have a different impact on the intensity of adoption (Sevier and Lee, 2004). The output from the model showed the level of significance and interaction of the dependent variable i.e., the SOCETs, and the explanatory variables, whether negative or positive and whether it was significant. The number of SOCETs adopted by farmers was treated individually and a Probit model was used.

The probability of a farmer adopting SOCETs is given by the expected benefits $I_{b}^{*}$ against the expected costs of not adopting the SOCETs, $I_{c}^{*}$. However, $I_{b}^{*}$ and $I_{c}^{*}$ are latent variables. The actual adoption of SOCETs, I, I = 1 if $I_{c}^{*}$*>*$I_{b}^{*}$ and I = 0 if $I_{c}^{*}$ ≤ ∗$I_{b}^{*}$. Adoption of $Y_{ij}$ can therefore be denoted as shown in Eq. (3):

(3) $$Y_{ij}=Z\alpha -\ddot{\upsilon }$$

Where $Y_{ij}$ is the SOCETs adoption dummy, Z is a vector of the independent variables affecting adoption of SOCETs and respective coefficients α and ϋ are an error term. The general Probit model of adopting SOCETs is therefore specified as shown in Eq. (4):

(4) $$Y_{ij}=\beta_{j}+\beta_{1}\chi_{ij}+\varepsilon_{ij}$$

where $Y_{ij}$ represents the SOCETs technology adoption, *i* is the index for household, *j* represents the ward or Pastoral area (*kebele*), $X_{ij}$ represents household characteristics, $\beta_{j}$ are ward or Pastoral area fixed effects and $\varepsilon_{ij}$ is the random error (Adusumilli and Wang, 2018).

#### 2.5.1 Descriptive results: plot level characteristics

Farmers worked on plots whose size ranged between 0.0025 and 6.18 acres. The slope of the plot ranged between 0.11 to 20.8°. Distance to the plots ranged from zero for those who lived on their farms to 210 walking minutes. 92% of the respondents perceived their soil to be fertile, 24% of the respondents perceived erosion to be a problem in their plots while 16% of the respondents lacked tenure security. Distance to urban market ranged between 7 and 240 walking minutes.

#### 2.5.2 Descriptive results: household characteristics

Six per cent of the households were female-headed. Gender affects the adoption of SOCETs in that there is high adoption of manure (61%) and fertilizer (98%) in female-headed households. The average household size is seven members. A household having many members affects the adoption of SOCETs especially, those that require high labour like manure where 94% adoption is seen in households with large sizes and fertilizer where 98% adoption is seen in households with many members. The majority of the household's heads are not employed but work on their farms as their main source of income while the average number of years that households have been involved in farming is 26 years.

#### 2.5.3 Descriptive results: biophysical factors

The average slope of the plot is 3.7° with the minimum slope being 0.11° and the maximum slope being 20.8°. Slope influences the adoption of fertilizer, manure and residue management. This is because it is directly related to soil erosion and farmers will adopt fertilizer and manure at a low rate if they suspect erosion will carry the inputs but adopt residue management to reduce erosion in high slopes. At an average slope of 3.7°, 95% of farmers have adopted fertilizer while 43% have adopted manure. 74% of the farmers perceive their plots to be susceptible to erosion and 91% perceive that their plots to be fertile. From these data, the mean rainfall received is 1442 mm with a minimum of 1105 mm and a maximum of 1828 mm.

### 2.6 Socioeconomic factors

86% of households have to tenure security or own land title deeds with mean plot sizes of 0.6 acres while the distance to the plots averages at 17 minutes. The average walking distance to the market is 89 minutes. 98% of households owned livestock, this was important in the adoption of manure as livestock are the main source of manure and crop residue management, which in some cases is fed to animals. 28% of households had access to credit. 57% of households were members of farmers' groups or associations and 57% of households had access to extension services. 19% of the household heads had attained secondary education. Households’ years in farming ranged from 2 to 60 years while the size of the household ranged between 1 and 13 members.

### 2.7 Testing multicollinearity

The variance inflation factor (VIF) was used in testing the multicollinearity among the explanatory variables. VIF assesses how much the variance of an estimated regression coefficient increases if your predictors are correlated. For all variables, the VIF was less than three indicating less collinearity.

## 3 Results

### 3.1 The extent of adoption of SOCETs

Survey results showed differences and relationships in adoption across the spatial divisions. This provides an essential insight into what economic incentives are required by farmers to adopt multiple SOCETs. The extent of SOCETs adoption, therefore, varies greatly across the two watersheds. Fertilizer is the most adopted technology having 89% of the plots in Azuga-Suba and 99% plots in Yesir having fertilizer application. Manure had 28% and 56% of the plots in Azuga-Suba and Yesir respectively having its application while residue management has been adopted in 37% and 26% of the plots in Azuga-Suba and Yesir respectively.

### 3.2 Probit model results on factors affecting adoption of SOCETs

The Probit econometric model was run on Stata separately for each response variable (SOCET) and on each Pastoral area. The following dependent variables were run, fertilizer, manure, and residue management. Factors affecting the adoption of these technologies were comparatively dissimilar suggesting that the adoption of SOCETs was heterogeneous.

The adoption of manure was significant in Ambercho Wasere, Bondenna, Bucha and Gerba Findide PAs in Azuga suba watershed as well as in Gulim, Jib Gedel, Tengeha and Wadra PAs in Yesir watershed (Table 2).

**Table 2:** Probit model regression results for the variables that affect the probability of adoption of manure in Ethiopia.

		Manure							
		Ambercho Wasere	Bondenna	Bucha	Gerba Findide	Gulim	Jib Gedel	Tengeha	Wadra
		Coef.	Coef.	Coef.	Coef.	Coef.	Coef.	Coef.	Coef.
Tenure security	Coef.	0.434	-0.22	-0.677	0	1.663∗∗∗	0.485∗∗	0.720∗∗	1.037∗∗∗
	Std. error	0.54	0.286	0.511	omitted	0.389	0.203	0.310	0.245
Distance to plot		-0.198∗∗∗	0.012	-0.072∗∗	-0.089∗∗∗	-0.003	0.040∗∗	-0.044∗∗∗	
		0.064	0.013	0.028	0.029	0.003	0.018	0.012	
Distance to market		0.048∗∗∗	0.0038	0.004	-0.057∗∗∗	-0.035∗∗∗	0.006∗	-0.073∗∗∗	0.005∗∗∗
		0.012	0.011	0.005	0.010	0.006	0.004	0.019	0.003
Slope of the plot		-0.4∗∗∗	-0.159∗∗	-0.052	-0.08∗∗	1.49∗∗∗	0.039	0.86∗∗	0.77∗∗∗
		0.104	0.074	0.138	0.041	0.200	0.034	0.351	0.296
Access to extension		-2.225∗∗∗	0	0	0.020	0	0.661∗∗∗	0	2.506∗∗∗
		0.618	omitted	omitted	0.301	omitted	0.202	omitted	0.332
Education level		0.329∗	0.104	0.100	-0.154	-0.051	0.114	-0.231	0.045
		0.178	0.106	0.257	0.162	0.081	0.073	0.148	0.107
Household size		0.206∗∗	-0.375∗∗∗	-0.565∗∗∗	0.426∗∗∗	0.184∗∗∗	0.092	0.42∗∗∗	0.082
		0.105	0.067	0.099	0.133	0.058	0.073	0.109	0.06
Farming experience		0.079∗∗∗	0.001	-0.011	-0.041∗∗	0.011	-0.006	-0.049∗∗∗	0.001
		0.025	0.013	0.015	0.020	0.008	0.006	0.017	0.010
Access to credit		0	0	0	0	0.401∗∗	0.059	-0.151	0.770
		omitted	omitted	omitted	omitted	0.195	0.193	0.338	0.296
Farmer groups membership		1.307∗	0.269	0	-1.926∗∗∗	0	-0.979∗∗∗	4.45∗∗∗	2.506
		0.786	0.331	omitted	0.681	omitted	0.199	0.760	0.332
Soil erosion		1.124∗∗∗	-1.066∗∗∗	0.024	0.332	-0.078	-0.458∗∗	-0.706∗	0.267
		0.389	0.221	0.289	0.416	0.231	0.183	0.374	0.329
Plot size		-0.729	0.347	-1.291	0.461	-0.557	-2.727∗∗	7.602∗∗∗	0.032
		1.00	0.553	2.142	0.377	0.215	1.369	2.259	0.745
Annual rainfall		0.121∗∗∗	-0.021	0.133∗∗∗	0.086∗∗∗	0.0582∗∗∗	0.002	-0.351∗∗∗	0.082∗∗∗
		0.040	0.041	0.020	0.021	0.014	0.002	0.071	0.017
Plot fertility		0	-0.720∗∗∗	-0.919∗∗∗	-0.255	0.058∗∗∗	0.175	-1.220∗∗	0
		omitted	0.265	0.324	0.507	0.014	0.243	0.492	omitted
Livestock ownership		0	0	0	0	0	2.196∗∗∗	0	0
		omitted	omitted	omitted	omitted	omitted	0.400	omitted	omitted

Note. ∗p < 0.10, ∗∗p < 0.05, ∗∗∗p < 0.01. The standard error is at the bottom of the coefficient. *N= 380 n*_*plots*_*= 2610*.

The adoption of fertilizers was significant in Ambercho Wasere, Bondenna and Gerba Findide, pastoral areas of Azuga-Suba watershed (Table 3).

**Table 3:** Probit model regression results for the variables that affect the probability of adoption of fertilizer in Ethiopia.

	Inorganic fertilizer		
	Ambercho Wasere	Bondenna	Gerba Findide
	Coef.	Coef.	Coef.
Distance to plot	1.279∗∗∗	0.002	0.033
	0.403	0.017	0.029
Distance to market	0.049∗∗∗	-0.024∗	0.029∗∗∗
	0.015	0.014	0.007
Slope of the plot	-0.435∗∗∗	-0.285∗∗∗	-0.086∗
	0.116	0.110	0.046
Access to extension	-1.685∗∗∗	0	0.415
	0.572	omitted	0.380
Plot size	4.991∗∗	-3.244∗∗∗	1.882∗∗∗
	2.887	0.666	0.662
Household size	0.316∗∗	0.026	-0.166∗∗∗
	0.163	0.078	0.064
Plot fertility	2.833∗∗∗	0.491	0.842∗
	0.633	0.313	0.483
Soil erosion	2.771∗∗∗	-0.465∗	0.022
	0.834	0.259	0.436
Annual rainfall	-0.113∗∗∗	-0.199∗∗∗	0.019
	0.035	0.070	0.019
Farming experience	-0.034	0.061∗∗∗	-0.017
	0.035	0.019	0.0183

Note. ∗p < 0.10, ∗∗p < 0.05, ∗∗∗p < 0.01. The standard error is at the bottom of the coefficient. *N= 380 n*_*plots*_*= 2610*.

The adoption of proper residue management was significant in Ambercho Wasere, Bondenna, Bucha and Gerba Findide pastoral areas in Azuga-Suba watershed as well as in Gulim, Jib-Gedel, Tengeha and Wadra pastoral areas in Yesir/Bure watershed (Table 4).

**Table 4:** Probit model regression results for the variables that affect the probability of adoption of residue management in Ethiopia.

	Residue management							
	Ambercho Wasere	Bondenna	Bucha	Gerba Findide	Gulim	Jib Gedel	Tengeha	Wadra
	Coef.	Coef.	Coef.	Coef.	Coef.	Coef.	Coef.	Coef.
Tenure security	-0.666	-1.168∗∗	3.431∗∗	-0.926	0.194	0.118	0.108	0.309
	0.563	0.597	1.349	0.961	0.214	0.183	0.359	0.354
Distance to plot	0.038∗∗	-0.033	0.334∗∗∗	-0.0191	0.004∗∗	-0.024∗∗	0.0005	-0.02∗∗
	0.015	0.026	0.127	0.015	0.002	0.012	0.01	0.012
Distance to market	-0.006∗	-0.031∗	-0.049∗∗∗	0.002	-0.005∗∗	0.007∗∗	-0.008	-0.002
	0.004	0.017	0.016	0.004	0.002	0.003	0.007	0.006
Slope of the plot	0.016	-0.417∗∗∗	-0.237	-0.15	0.200∗∗	0.060∗∗	-0.309	0.0664
	0.031	0.110	0.499	0.024	0.097	0.029	0.287	0.104
Plot size	1.656∗∗∗	-4.559∗∗∗	-8.932∗∗	1.047∗∗∗	0.339∗	-3.216∗∗	-0.894	-4.5∗∗
	0.509	1.589	4.544	0.290	0.185	1.377	1.358	1.965
Household size	0.103∗∗∗	-0.586∗∗∗	0.536	-0.188∗∗∗	-0.008	-0.052	0.3∗∗∗	-0.095
	0.039	0.105	0.375	0.049	0.038	0.055	0.122	0.104
Farming experience	0.003	-0.009	-0.0549	0.028∗∗∗	-0.02∗∗∗	-0.011	0.0002	0.015
	0.010	0.022	0.044	0.011	0.007	0.007	0.014	0.011
Farmer groups membership	-2.917∗∗∗	-1.008∗	0	-0.880∗∗∗	1.536∗∗∗	0.458∗∗∗	1.5∗∗∗	-1.1∗∗∗
	0.676	0.525	omitted	0.245	0.517	0.177	0.373	0.297
Access to credit	1.140	0	0	-0.812∗∗	-0.4∗∗∗	0.008	-0.7∗∗	-0.086
	0.646	omitted	omitted	0.336	0.157	0.172	0.290	0.298
Plot fertility	-0.694∗∗	0	0.687	0.262	0	-0.215	0.767	0
	0.286	omitted	0.729	0.380	omitted	0.201	0.754	omitted
Soil erosion	0.423∗∗	-1.819∗∗∗	2.169∗∗∗	-0.158	-0.231	-0.6∗∗∗	-0.659	-0.564
	0.197	0.471	0.835	0.252	0.214	0.183	0.428	0.603
Education level	0.030	-0.001	-0.178∗	0.071∗∗∗	0.036∗	-0.008	-0.024	-0.063∗
	0.022	0.029	0.096	0.023	0.018	0.018	0.030	0.034
Annual rainfall	0.012	0.053	-0.480∗∗∗	0.030∗∗	-0.1∗∗∗	-0.004∗	-0.037	-0.026
	0.009	0.0604	0.157	0.013	0.013	0.002	0.025	0.015
Access to extension	0.363	0	0	-0.585∗∗∗	-0.929	-0.0137	0.095	0.886
	0.230	omitted	omitted	0.222	0.902	0.184	0.399	0.656

Note. ∗p < 0.10, ∗∗p < 0.05, ∗∗∗p < 0.01. The standard error is at the bottom of the coefficient. *N= 380 n*_*plots*_*= 2610*.

## 4 Discussion

### 4.1 Factors influencing adoption of SOCETs

#### 4.1.1 Manure

The likelihood of the adoption of manure in Ambercho Wasere was positively influenced by distance to market, education level of the household head, household size, farming experience, membership in farmer groups, soil erosion perception and annual rainfall (Table 2). This is evident where 100% of the adopters lived far from the market, had a farming experience of over 17 years and lived in areas that received rainfall of over 1120 mm. It is also evident where 90% of the adopters had educated household heads, 95% were members of farmer groups and 70% perceived erosion to be a problem in their plots. On the other hand, the likelihood to use manure was influenced negatively by extension, the slope of the plot and distance to the plot. This is evident where 100% of the adopters lived closer to their plots, 95% lacked access to extension and 13% of the adopters lived in areas with high slopes.

In Bondenna, the slope of the plot, household size, plot fertility perception and soil erosion perception negatively influenced manure adoption (Table 2). This is evident where, only 20% of the adopters lived on high slopes, only 10% of adopters had household sizes of more than six members, 8% of adopters perceived their soil to be fertile and 18% of the adopters perceived to have erosion problem. In Bucha, manure adoption was influenced positively by annual rainfall and negatively by household size, distance to the plot and plot fertility perception. This is evident where 100% of the adopters lived in areas receiving rainfall greater than 1120 mm. On the other hand, only six per cent of the adopters had more than six household members while only 8% lived far from their plots and only 18% perceived their plots to be fertile. In Gerba Findide, manure adoption was influenced positively by household size and annual rainfall. This is evident where 100% of the adopters lived in areas that received rainfall of more than 1120 mm while 94% had more than six household members. On the other hand, manure adoption was influenced negatively by distance to plots, distance to market, the slope of the plot, farmer group membership and farming experience. This is evident where only 8% of the adopters lived further away from their plots, 47% of the adopters lived 60 minutes away from the market, only 11% lived in sloppy areas, 40% were farmer group members and only 7% had a farming experience of more than 10 years.

In Gulim, adoption of manure was influenced positively by tenure security, the slope of the plot, household size, and annual rainfall and access to credit. This is evident where 93% of the adopters had tenure security, 91% had plots in areas with slopes greater than 2.5°, 93% of adopters had household sizes of more than 6 members, 53% of the adopters had access to credit and 100% of the adopters are located in areas receiving rainfall of over 1215 mm. On the other hand, manure adoption was negatively influenced by distance to market and plot sizes. This is evident where only 1% of the adopters lived far from the market while 77% of the adopters had plot sizes of less than 0.25 ha. In Jib Gedel, adoption of manure was influenced positively by tenure security, distance to plot and distance to market, extension and livestock ownership. This is evident where 76% of the adopters had tenure security, 72% adopters lived more than 10 minutes away from their plots, 97% lived more than 60 minutes from the market, 77% had access to extension and 98% owned livestock. On the other hand, manure adoption was negatively influenced by farmer group membership, soil erosion and plot sizes. This is evident where, 44% of the adopters were in farmer groups, 32% of the adopters perceived erosion was a problem and 98% had plot sizes of less than 0.25°.

In Tengeha, adoption of manure was influenced positively by tenure security and slope of the plot, household size, membership in farmer groups and plot size. This is evident where 76% of the adopters had tenure security, 99% had plots in slopes of more than 2.5°, 90% had more than six family members, 67% were members of farmer groups and 75% had plot sizes greater than 0.25 ha. It was negatively affected by distance to the plot, distance to market, education level, farming experience, plot fertility perception, erosion perception and annual rainfall. This is evident where only 15% of adopters live less than 10 minutes away from their plots, 35% of the adopters lived more than 60 minutes from the market and 28% had a high level of education. In addition to this, 33% had a farming experience of more than 10 years, 3% perceived their plots to be fertile, 16% had soil erosion problems and 1% were located in areas receiving over 1215 mm of rainfall. In Wadra, the adoption of manure was influenced positively by tenure security, market distance, access to credit, membership in farmer groups and annual rainfall. This is evident where 78% of the adopters had tenure security, 63% lived more than 60 minutes from the market, 73% has access to credit, were in farmer groups and 100% lived in areas receiving over 1215 mm of rainfall.

#### 4.1.2 Fertilizers

The likelihood to use fertilizers in Ambercho Wasere was positively influenced by distance to the plot, distance to market, plot size, household size, plot fertility perception and soil erosion (Table 3). This is evident where 73% of adopters have plot sizes of more than 0.25 ha, 51% of adopters lived more than 60 minutes away from the market, 89% of adopters perceived their plots to be fertile and 99% of the adopters perceived erosion to be a problem. It was influenced negatively by extension, the slope of the plot and annual rainfall. This is evident where 64% of the adopters had no access to extension, 99% of the adopters have plots with a slope less than 2.5° and 99% of the adopters are located in areas receiving rainfall less than 1205 mm. In Bondenna, it was positively influenced by farming experience. This is seen where 91% of adopters had a farming experience of over 10 years. It was negatively influenced by distance to market, the slope of the plot, annual rainfall, plot size, and erosion perception. This is evident where 100% of adopters lived less than 60 minutes away from the market, 25% owned plots in slopes greater than 2.5°, 89% owned plot sizes less than 0.25 ha, 74% perceived erosion to be a problem, 11% were located in areas receiving rainfall above 1205 mm. In Gerba Findide, adoption was influenced positively by distance to market and plot fertility perception. This is evident where 89% of adopters lived more than 60 minutes away from the market and 97% perceived their plots to be fertile. It was influenced negatively by the slope of the plot and household size. This is evident where only 2% of the adopters owned plots with a slope greater than 2.5° and 1% had household sizes of more than six members.

#### 4.1.3 Residue management

The likelihood to adopt proper residue management in Ambercho Wasere was positively influenced by distance to plot, plot fertility perception and soil erosion perception, plot size and household size (Table 4). This is evident where 98% of the adopters lived more than 30 minutes away from their plots, 100% owned plot sizes greater than one ha, 77% had household sizes of more than 6 members, 95% perceived their plots to be fertile and 21% perceived erosion to be a problem. It was influenced negatively by distance to market and farmer group membership. This is evident where 66% of adopters lived less than 60 minutes away from the market and 98% were not members of farmer groups. In Bondenna residue management adoption was negatively influenced by tenure security, distance to market and farmer group memberships, soil erosion perception, household size, plot size and slope of the plot. This is evident where only 2% of adopters had tenure security, 85% lived less than 60 minutes away from the market and 29% had plot+s with a slope of more than 2.5°. In addition to this, 88% had household sizes of more than 6 members, 78% had plot sizes greater than 0.25 ha, 17% perceived erosion to be a problem and 2% were not members of farmer groups.

In Bucha adoption was influenced positively by tenure security, distance to plot and soil erosion perception. This is evident where 97% of adopters had tenure security, 100% lived more than 30 minutes away from their plots and 55% perceived erosion to be a problem. It was negatively influenced by distance to the market and annual rainfall, level of education and plot size. This is evident where 74% of adopters lived less than 60 minutes away from the market, only 38% had a high education level, 99% had plot sizes less than 0.25 ha and 100% were located in areas receiving less than 1205 mm of rainfall. In Gerba Findide, adoption was influenced positively by annual rainfall, education level, plot size and farming experience. This is evident where 41% of adopters had high education level, 99% had plot sizes greater than 0.25 ha, 95% had a farming experience of over 10 years, 70% were located in areas receiving rainfall more than 1215 mm. It was influenced negatively by access to extension, household size and access to credit and farmer group membership. This is evident where 33% of adopters had access to extension, 7% had household sizes of more than six members, 94% had no access to credit and 91% were not members of farmer groups.

In Gulim, adoption of residue management is influenced positively by distance to plot and slope of the plot, level of education, plot size and farmer group membership. This is evident where 70% of adopters live more than 30 minutes away from their plots, 96% had plots with a slope greater than 2.5°, 71% had a high education level, 100% had plot sizes greater than 0.25 ha and 97% were members of farmer groups. It was influenced negatively by distance to market, farming experience, access to credit and annual rainfall. This is evident where 67% of adopters lived less than 60 minutes away from the market, 47% had a farming experience of over 10 years, 42% had access to credit and 44% were located in areas receiving rainfall more than 1215 mm. In Jib-Gedel, the adoption of residue management was influenced positively by market distance and plot slope and farmer group membership. This is evident where 77% of adopters lived more than 60 minutes away from the market, 77% had plots with a slope of more than 2.5° and 63% were members of farmer groups. It was influenced negatively by distance to plot and plot size, soil erosion perception and annual rainfall (Table 4). This is evident where 100% of adopters lived less than 30 minutes away from their plots, 78% had plot sizes less than 0.25 ha, 17% perceived erosion to be a problem and 100% of adopters are located in areas receiving rainfall more than 1215 mm. In Tengeha, the adoption of residue management was influenced positively by household size and farmer group membership. This is evident where 87% of adopters had household sizes of more than six members and 90% were members of farmer groups. It was influenced negatively by access to credit services (Table 4). This is evident where 59% of adopters had no access to credit. In Wadra, adoption of residue management was influenced negatively by distance to plot, market distance and plot size and farmer groups membership (Table 4). This is evident where 89% of adopters live less than 30 minutes away from their plots, only 27% of the adopters lived more than 60 minutes away from the market, 2% had plot sizes more than 0.25 ha and 77% were not members of farmer groups.

### 4.2 Constraints to the adoption of sustainable land management technologies

#### 4.2.1 Manure

Distance to market, farming experience, membership in farmer groups or associations, soil erosion perception, annual rainfall, distance to the plot and soil fertility perception constrain the adoption of manure in both Yesir and Azuga-Suba watersheds. In addition, plot size constrains adoption of manure in Yesir watershed while the slope of the plot, access to extension and household size constrains adoption of manure in Azuga-Suba watershed.

Reduction in the use of manure is because farmers who are educated know how to use both manure and fertilizer to increase production (Mustafa-Msukwa et al., 2011). Short distances to the market are expected to reduce the relative costs and availability of inputs while long distances to the market would raise fertilizer costs, which would tend to make farmers turn to manure (Waithaka et al., 2007). Closeness to urban markets, therefore, ensures that farmers can easily access inorganic fertilizers and therefore leads to limited use of manure (Mwangi, 1996). It is expected that, experienced farmers are more likely to adopt manure use (Liu et al., 2017). This is because farming experience equips farmers with the right knowledge on the use of both manure and fertilizers in the right amount to increase production (Waithaka et al., 2007). This leads to the use of less amount of manure. Being a member of farmer groups and associations leads to improved knowledge to various soil enhancement technologies giving farmers many options (Mwangi, 1996). Farmers may decide to adopt fertilizers, which requires less labour compared to manure and provides nutrients faster than manure, which is long term. Tenure security inspires farmers to invest in permanent land management systems including the addition of manure (Kassie, 2016). Large-sized plots and plots located far from the farmers’ homes discourages the application of manure. This is because they are labour intensive due to transportation and the addition of manure which is bulky (Waithaka et al., 2007; Giller et al., 2006). Plots susceptible to soil erosion discourage the addition of manure due to the risk of losing it through runoff (Larney and Janzen, 1996). Compared to the unfertile plots, there is a laxity in addition of manure on fertile farms. High annual rainfall is associated with erosion constraining farmers from adoption to manure use (Larney and Janzen, 1996) while low annual rainfall constrains the adoption of manure. This is because low rainfall especially at the start of the season tends to cause the manure to scorch the crops (Paulus, 2015). A household with more members depending on the farm tends to use less fertilizer and more manure. This is because these households are more concerned about food provision for the household instead of other income-related objectives (Waithaka et al., 2007). The probability of adopting manure decreases with an increase in slope steepness. According to Clay et al. (1998), farmers tend to use manure on flat plots as compared to steep plots because of severe runoff associated with high slopes. Adoption of manure decreases with distance to the plots (Ketema and Bauer, 2011; Nkonya et al., 2008). This is because of the bulky nature of manure, which requires transporting to distant plots.

#### 4.2.2 Fertilizer

The constraints to the adoption of fertilizer use in Ethiopian watersheds include soil erosion perception, farming experience, access to extension, the slope of the plot, annual rainfall, and distance to markets, plot size and household size. Extension services raise farmers' awareness of the benefits of fertilizers (Eba and Bashargo 2014). This increases the adoption of fertilizer use. However, the extension services also provide farmers with technological options that can be used alongside manure. This leads to a reduction in the use of fertilizers as farmers get more knowledge on the negative effects of using these fertilizers on the soil and soil micro-organisms (Geisseler and Scow, 2014). According to Waithaka et al. (2007) and Gebresilassie and Bekele (2015), longer distances to urban markets increase the cost of fertilizer and the time needed to access them discourages their use. For most smallholder farmers in Africa, purchasing power is low and therefore with increasing plot size, there is a decrease in the ability to afford the amount of fertilizer required (Akpan et al., 2012).

Farming experience is known to improve farmers’ skills (Eba and Bashargo 2014) encouraging the adoption of various SOCETs and therefore, lack of experience is a constraint to adoption. However, with long term farming experience, farmers have more options on top of fertilizer adoption such as the use of manure and proper residue management. Rain-fed agriculture is mainly associated with risks of low rain (Tsehaye, 2008), this constrains the adoption of the use of fertilizer as farmers fear making losses whenever there are low rains. Where farmers have relatively fertile farms, they tend to be reluctant in adding fertilizers (Tsehaye, 2008; Onyenweaku et al., 2007; Marenya et al., 2008). According to Tchale et al. (2004), large household sizes result in large food demand leading to households suffering from chronic food shortages. These households, therefore, lack financial resources to purchase fertilizers as their money is used mostly to purchase food. Sloping plots are more susceptible to soil erosion resulting in smaller yields as compared to flat land (Aemro and Musa, 2016). Farmers, therefore, prefer investing in flatter plots than plots on the slope since they provide higher yields (Clay et al., 1998). Farmers, therefore, do not apply fertilizers on slopping plots due to erosion which lead to losses (Tadesse, 2014). Farmers with access to agricultural extension and those who are members of organizations are more likely to use fertilizer on their plots (Birungi, 2007; Nkonya et al., 2008). This is because they form important channels through which agricultural education and information are disseminated to farmers (Ketema and Bauer, 2011).

#### 4.2.3 Residue management

Distance to the market, plot size, soil erosion perception, level of education of household head, membership in farmer groups or associations, access to credit, farming experience and annual rainfall constraints the adoption of residue management in Azuga-Suba and Yesir watershed. In addition to this, distance to plot and soil fertility perception constraints adoption of residue management in Yesir while tenure security, household size, the slope of the plot and access to extension constraints adoption in Azuga-Suba.

Closeness to markets reduces the costs of transporting farm outputs and inputs to and from the markets thereby raising profitability and lowering the input costs (Mponela et al., 2016). Farmers living close to these markets will therefore sell the residue for other uses to increase their profits with the notion that they can access fertilizers at a cheaper rate as a substitute for increasing soil fertility. Small-sized plots, due to continuous divisions exacerbate land tenure problems (Mugure et al., 2013) discouraging residue management. Lack of tenure security discourages residue management as farmers want to harvest every part of the crop to attain maximum benefits. With adequate crop residues use, soil erosion in a plot is greatly reduced and water management enhanced (Unger et al., 1991). However, if little amounts of residues are used, they can be carried away by erosion leading to losses. This discourages adoption. Farmers who are members of groups or associations are more likely to adopt residue management (Liu et al., 2017). This is because, in groups, farmers are exposed to a wide range of new information and allow individuals to learn and share information on agricultural inputs and marketing (Odendo et al., 2011). Farmers are also able to share experiences and knowledge enabling farmers to obtain the knowledge of other technologies such as composting which encourage the use of residues to make compost manure. Credit improves the ability of farmers to purchase inputs as well as pay for labour (Gachene and Wortmann, 2007). Lack of access to credit leads to the poor purchasing power of farm inputs, animal feeds and other commodities such as fuel and building materials leading to farmers substituting these inputs with residues (Jassogne et al., 2013). As larger households provide farmers with more dependable access to inexpensive labour (Kammer, 2014) the demand for food is also higher. This brings about a requirement to use every piece of biomass to bring food to the table including selling residues for fodder (Tittonell et al., 2009). The extension may enhance farmers' knowledge and understanding of residue management, therefore, increasing adoption but may also increase the knowledge of other land management options such as manure which encourages feeding of residues to livestock in a bid to increase manure production (Fisher et al., 2018). According to International Institute of Rural Reconstruction (IIRR), (1995), residues are difficult to spread on highly sloping slopes and in some cases being washed away by water discouraging adoption. Long distances to plots discourage the transfer of residues for other uses compared to plots, which are closer to home (Kelsey, 2013). Therefore, having plots close to home constraints adoption. Farmers’ perception that their plots are fertile constraints adoption of the use of residues for fertility improvement discouraging adoption (Odendo et al., 2011).

## 5 Conclusion and recommendations

The results demonstrate that the extent of adoption of SOCETs is influenced by several variables such as group membership to local institutions, credit constraints, head access to basic education, distance to markets, rainfall, plot slope farming experience, soil erosion, soil fertility, plot sizes, household size, tenure security, extension and distance to plot. The significant importance of constraints relating to social capital (such as membership in farmer groups and associations, credit, household head education, livestock ownership, distance to markets, rainfall, plot slope and sizes, household sizes, extension etc.) on the adoption of SOCETs suggests that there is a need for establishing and strengthening local institutions and service providers to accelerate and sustain SOCETs adoption.

Local organizations assume critical roles of enriching farmers with timely information, providing inputs (e.g. labour, credit, insurance) and technical assistance. The significance of accessing credit is tied to its influence on the ability to purchase inputs (improved seed and fertilizer) while being a member of farmer groups or associations ensures that a farmer is able or may get subsidies or free inputs from the agricultural institutions. Livestock ownership ensures that farmers have some manure by the start of a season therefore, ensuring every farmer owns at least one or two improved breeds and improved forage legumes would ensure that there is an increase in products sourced from livestock including manure. Rainfall effects on the adoption of SOCETs are centred on slope and erosion for areas receiving high rainfall. Rainfall disturbance in addition to inorganic fertilizer results from the scorching of plant roots due to inadequate rainfall resulting from less water to dissolve the chemicals. Rainfall forecasting is important in ensuring proper timing and distribution. In addition to this, the use of SOCETs is associated positively with the farmer's access to extension, education and involvement in associations. This is because these are associated with personal development by providing information to the farmers. This proposes an investment in a proper and working extension service provision, farmers training centres and the formation of farmer groups with linkages to the government and non-governmental institutions that can establish a positive effect on the adoption of SOCETs. Investment in rural state-funded education will encourage the adoption of SOCETs and practices.

## Declarations

### Author contribution statement

All authors listed have significantly contributed to the development and the writing of this article.

### Funding statement

This work was supported by the Federal Ministry of Economic Cooperation and Development (BMZ/GTZ) Contract no.: 81206681. Project no.: 16.7860.6e001.00.

### Data availability statement

Data associated with this study has been deposited at Survey data on factors that constrain the adoption of soil carbon enhancing technologies in Ethiopia: https://doi.org/10.6084/m9.figshare.11830887.

### Declaration of interests statement

The authors declare no conflict of interest.

### Additional information

No additional information is available for this paper.

## Appendix A Supplementary data

Questionnaire Questionnaire
