# Supplementary material for: Factors constraining the adoption of soil organic carbon enhancing technologies among small-scale farmers in Ethiopia
Source: Heliyon. 2021 Nov 27;7(12):e08497. doi: 10.1016/j.heliyon.2021.e08497 (PMC8646156; doi:10.1016/j.heliyon.2021.e08497)
Supplement: Questionnaire [file mmc1.docx]

**QUESTIONNAIRE PREPARED FOR HOUSEHOLD INTERVIEW**

**Prepared for a Research on**

**Adoption of carbon enhancing practices**

**International Institute of Tropical Crops (CIAT)**

**Fekadu Gelaw**

**Department Of Agricultural Economics**

**Haramaya University**

**Interviewed by: Date / /2018 Time : Signature**

**Checked by: Date / /2018 Time : Signature**

| Zone |  | Woreda |  |
| --- | --- | --- | --- |
| Name of PA |  | Name of the village /Gotte/ |  |
| Household Unique ID |  | Starting time |  |
| GPS coordinates | Northing [____________] | Easting [____________] | Altitude [______ ] m.a.s.l |

**Section I. Household characteristics**

**1. General**

- 1. Name of the household head [ **________________________________**]
  2. Age [________] (years)
  3. Gender of the household head [______] **(Code-B1)**
  4. Were you born here? [_______] **(Code-A)** If NO, how many years have you lived here? [________] (years)
  5. How many years have you been involved in farming? [________] (years)

***NB****: Question 1.6 to 1.7 need to be answered if and only if the household head is* ***NOT*** *the respondent*

- 1. Name of the respondent [_________________________________] Age [________] (years)
  2. Gender of the respondent [______] (Code-B1)

**2. Detail about household demography**

How many people live and eat in the household? Please tell us about each member of the household

| P.I.D. | | 2.1. | | | 2.2 | | | 2.3 | 2.4 | | | 2.5 | | 2.6 | | | | | 2.7 | 2.8 | |
| --- | --- | --- | --- | --- | --- | --- | --- | --- | --- | --- | --- | --- | --- | --- | --- | --- | --- | --- | --- | --- | --- |
|  |  | Name (First name only) | | | Age (yrs) | | | Sex  (Code-B1) | Marital status (Code-B2) | | | Relationship (Code-B3) | | Education level | | | | | Main occupation (Code-B5) | Does the person participate in farming activities? (provide labor?) (Code-A) | |
|  |  |  |  |  |  |  |  |  |  |  |  |  |  | Literacy level  (Code-B4) | | Grade completed | | |  |  |  |
|  | |  | | |  | | |  |  | | |  | |  | |  | | |  |  | |
|  | |  | | |  | | |  |  | | |  | |  | |  | | |  |  | |
|  | |  | | |  | | |  |  | | |  | |  | |  | | |  |  | |
|  | |  | | |  | | |  |  | | |  | |  | |  | | |  |  | |
|  | |  | | |  | | |  |  | | |  | |  | |  | | |  |  | |
| Yes/No (Code-A) | | | (Code-B1) | | | (Code-B2) | | | | (Code-B3) | | | (Code-B4) | | | | **(Code-B5)** | | | |  |
| 1 | Yes | | 1 | Male | | 1 | Married | | | 1 | Head | | 1 | | Illiterate | | 1 | Farmer | | |  |
| 2 | No | | 2 | Female | | 2 | Single | | | 2 | Wife/husband | | 2 | | Informal educ. | | 2 | Pastoralist | | |  |
|  |  | |  |  | | 3 | Divorced | | | 3 | Son/daughter | | 3 | | Formal schooling | | 3 | Craftsmen | | |  |
|  |  | |  |  | | 4 | Widow | | | 4 | Mother/Father | | 4 | | Vocational | | 4 | Trader | | |  |
|  |  | |  |  | | 5 | Not-together | | | 5 | Brother/Sister | | 5 | | Other, specify___ | | 5 | Daily-labor | | |  |
|  |  | |  |  | |  |  | | | 6 | Grandchild | |  | |  | | 6 | Petty business | | |  |
|  |  | |  |  | |  |  | | | 7 | Servants | |  | |  | | 7 | Private: skilled | | |  |
|  |  | |  |  | |  |  | | | 8 | Other, specify ____ | |  | |  | | 8 | Private: unskilled | | |  |
|  |  | |  |  | |  |  | | |  |  | |  | |  | | 9 | Public: skilled | | |  |
|  |  | |  |  | |  |  | | |  |  | |  | |  | | 10 | Public: unskilled | | |  |
|  |  | |  |  | |  |  | | |  |  | |  | |  | | 11 | Other, specify_______ | | |  |

2.9 How many of the above household members are attending school? In number Male [________] Female [________]

**3. Household wealth indicators**

3.1. Is the household’s house made of corrugated iron sheet? [________] **(Code-A)**

- 1. If yes to 3.1, what is the total size of the house? (i.e. number of corrugated iron sheets) [________] (zingo)

What is the size of other houses e.g. store, livestock pen, guest house etc [________] (number of corrugated iron/*zingo*)

- 1. How many TV sets does the household have? [________] (number of houses)
  2. How many radios does the household have? [________] (number of houses)
  3. How many sofa sets does the household have? [________] (number of houses)
  4. How many toilets does the household have? [________] (number of houses)
  5. How many of the household members have mobile apparatus? [________] (number of houses)
  6. How many motor pumps does the household have? [________] (number of houses)
  7. How many horse carts does the household have? [________] (number of houses)
  8. Does the household own any of the livestock? [________] **(Code-A)**

If the answer to question 3.10 is YES, fill the table, if NO skip to question 4

| No | Type of Livestock | 3.11 | 3.12 | 3.13 |
| --- | --- | --- | --- | --- |
|  |  | Number owned CURRENTLY | How many did you sell in the past 12 months? | Total revenue from sales (in Eth. Birr) |
| 1 | Bull/Ox |  |  |  |
| 2 | Cows/Heifer |  |  |  |
| 3 | Calves |  |  |  |
| 4 | Sheep/Goat |  |  |  |
| 5 | Horse/Mule |  |  |  |
| 6 | Donkey |  |  |  |
| 7 | Beehives |  |  |  |

4**. Household’s access to infrastructural services**

Tell us the walking distance in minute to the nearest infrastructure listed below

| No. | Infrastructure | 4.1. |
| --- | --- | --- |
|  |  | Nearest distance in walking minute |
| 1 | All weather motorable road |  |
| 2 | Winter motorable road |  |
| 3 | Asphalt road |  |
| 4 | Nearest rural market |  |
| 5 | Nearest town market |  |
| 6 | Nearest electricity |  |
| 7 | Nearest health post |  |
| 8 | Nearest health center |  |

**Section II. Plot level data**

**5. Description of plots of land owned and operated**

5.1. How many plots of land the household own? [_______] (*in number)*

5.2. How many plots of land the household operated last year in 2017 (Ethiopian 2009/10 production year) [_______] (*in number)*

Could you tell us in detail about the plots of land owned and operated by the household last year in 2017 (Ethiopian 2009/10 production year)

|  | 5.3 | 5.4 | 5.5 | 5.6 | 5.7 | 5.8 | 5.9 | 5.10 | 5.11 | 5.12 |
| --- | --- | --- | --- | --- | --- | --- | --- | --- | --- | --- |
| Plot ID | Unit  (Code-Ra1) | Plot size/area | Crop (Code-Rc) | Yield | | If irrigated, Crop type (Code-Rc) | Previous crop type (Code-Rc) | Number of years since the plot was left fallow | About the residue | |
|  |  |  |  | Unit (Code-Ra) | Qty. |  |  |  | What proportion was left in the plot (%) | For what purpose the rest was used? (Code-E) |
| 1 |  |  |  |  |  |  |  |  |  |  |
| 2 |  |  |  |  |  |  |  |  |  |  |
| 3 |  |  |  |  |  |  |  |  |  |  |
| 4 |  |  |  |  |  |  |  |  |  |  |
| 5 |  |  |  |  |  |  |  |  |  |  |
| 6 |  |  |  |  |  |  |  |  |  |  |
| 7 |  |  |  |  |  |  |  |  |  |  |
| 8 |  |  |  |  |  |  |  |  |  |  |
| 9 |  |  |  |  |  |  |  |  |  |  |
| 10 |  |  |  |  |  |  |  |  |  |  |

**Plot level detail data continued …..**

|  | 5.13 | 5.14 | 5.15 | 5.16 | 5.17 | 5.18 | 5.19 | 5.20 | 5.21 |
| --- | --- | --- | --- | --- | --- | --- | --- | --- | --- |
| Plot ID | Do you believe the plot is fertile (Code-A) | Soil type (Code-H) | Slope (Code-I) | How far is the plot from home (in walking minute) | Do you believe soil erosion is a problem at the plot (Code-A) | Tenure (Code-J) | If hired in/out, for how much (Eth. Birr) | Who manages the plot (Code-K) | Do you have land certificate? (Code-A) |
| 1 |  |  |  |  |  |  |  |  |  |
| 2 |  |  |  |  |  |  |  |  |  |
| 3 |  |  |  |  |  |  |  |  |  |
| 4 |  |  |  |  |  |  |  |  |  |
| 5 |  |  |  |  |  |  |  |  |  |
| 6 |  |  |  |  |  |  |  |  |  |
| 7 |  |  |  |  |  |  |  |  |  |
| 8 |  |  |  |  |  |  |  |  |  |
| 9 |  |  |  |  |  |  |  |  |  |
| 10 |  |  |  |  |  |  |  |  |  |

| (Code-A) | | **Reason (Code-C)** | | **Use of crop residue (Code-E)** | | **Fertility (Code-G) -** | | **Soil type (Code-H)** | | **Slope (Code-I)** | | **Tenure (Code-J) -** | | **Who manage the plot (Code-K)** | |
| --- | --- | --- | --- | --- | --- | --- | --- | --- | --- | --- | --- | --- | --- | --- | --- |
| 1 | Yes | 1 | No erosion problem | 1 | Cooking food | 1 | Fertile | 1 | Clay | 1 | Sloppy | 1 | Owned | 1 | Family member |
| 2 | No | 2 | Shortage of labor | 2 | Livestock feed | 2 | Not fertile | 2 | Loam | 2 | Plain | 2 | Rented in | 2 | Relative |
|  |  | 3 | Doubts its effectiveness | 3 | Construction |  |  | 3 | Sandy |  |  | 3 | Rented out | 3 | Other family member |
|  |  | 4 | Don’t know what to do | 4 | Soil fertility enhancement |  |  |  |  |  |  | 4 | Sharecropped in | 4 | Hired worker |
|  |  | 5 | No skills to implement it | 5 | Other, specify______ |  |  |  |  |  |  | 5 | Sharecropped out | 5 | Other, specify_____ |
|  |  | 6 | Other ______________ |  |  |  |  |  |  |  |  | 6 | Bought |  |  |
|  |  |  |  |  |  |  |  |  |  |  |  | **7** | Borrowed in |  |  |
|  |  |  |  |  |  |  |  |  |  |  |  | 8 | Borrowed out |  |  |

**6. Input use**

6.1. Did you use inorganic/organic fertilizer in any of the plots in 2017 (Ethiopian 2009/10 production year)? [________] (Code-A)

If your answer to question 6.1 is yes, tell us about your input uses

| Plot ID | 6.2 | 6.3 | 6.4 | 6.5 | 6.6 | 6.7 | 6.8 | 6.9 | 6.10 |
| --- | --- | --- | --- | --- | --- | --- | --- | --- | --- |
|  | Inorganic fertilizer use | | | | | Manure use | | | |
|  | Type **(Code-L)** | Unit (Code-Ra) | Qty | Price per unit (if bought) | Source **(Code-M)** | Plot ID | Unit (Code-Ra) | Qty | Price per unit (if bought) |
|  |  |  |  |  |  |  |  |  |  |
|  |  |  |  |  |  |  |  |  |  |
|  |  |  |  |  |  |  |  |  |  |
|  |  |  |  |  |  |  |  |  |  |
|  |  |  |  |  |  |  |  |  |  |

6.11. Where do you source your labor for farm activities? [______] **(Code-N)**

| Who constructed them?  (Code-L) | |  | Sources of inorganic fertilizer (Code-M) |  | Labor source  (Code N) |
| --- | --- | --- | --- | --- | --- |
| 1 | DAP | 1 | Private traders | 1 | Family labor only |
| 2 | Urea | 2 | Input suppliers | 2 | Hired labor only |
| 3 | NPS | 3 | Cooperative | 3 | Family and hired labor |
| 4 | Lime | 4 | GO/NGO | 4 | Collaborative labor (*Debo/Jige)* |
| 5 | Other, specify _________ | 5 | Other, specify _________ |  |  |

6.12. Do you or member of your household work for others to get income? [______] **(Code-A)**

**Section III. About carbon enhancing practices**

**7. Soil conservation and agroforestry activities**

7.1. Have you implemented any of the soil management practices (*refer to Code-Rf for the list of activities*) on any of your plots? [____] **(Code-A)**

7.2. If the answers in questions 7.1 above is YES, please tell us on plot basis about the specific activity you implemented so far by in the Table below

| No. | What soil management practices you implemented so far? **(Code-Rf)** | 7.3 | | | | 7.4 | 7.5 | 7.6 | 7.7 |
| --- | --- | --- | --- | --- | --- | --- | --- | --- | --- |
|  |  | In which plots did you apply the respective management practices? **(Plot codes)** | | | | Can you quantify the total activity implemented? | | How long have you been practicing it (in number of years) | For what purpose were they constructed/p? **(Code-O)** |
|  |  |  |  |  |  | Unit (Code-Ra) | Qty |  |  |
| 1 |  |  |  |  |  |  |  |  |  |
| 2 |  |  |  |  |  |  |  |  |  |
| 3 |  |  |  |  |  |  |  |  |  |
| 4 |  |  |  |  |  |  |  |  |  |
| 5 |  |  |  |  |  |  |  |  |  |

7.8. You mentioned that ____ of the plots are not-fertile (*see column 5.13 above*), did you do something to improve the soil fertility? [____] **(Code-A)**

7.9. If the answer to 7.8 is YES, what did you do to improve the soil fertility of the plots? [_________] **(Code-P)**

*Note: If the answer to the choice [****fertility improving (Code-P)]*** *is different from 1, go back to question 7.1 and revise its answer and also record the detail about the activity in table 7.2)*

7.9. If your answer to question 7.8 is NO, do you see any importance of implementing soil management practices to improve soil fertility of these plots? [____] **(Code-A)**

If the answer to 7.8 is YES, which **soil fertility management** practices you deem necessary for your plots? [___________] **(Code-Rf)**

7.11. What are the **challenges** that hindered you to do the practices you mentioned in question 7.9? [___________] **(Code-Q)**

| The purpose it was implemented (Code-O) | | Fertility improving activities (Code-P) | | Challenges (Code-Q) | |
| --- | --- | --- | --- | --- | --- |
| 1 | To improve soil fertility | 1 | Used inorganic fertilizer | 1 | Reduce production |
| 2 | To reduce soil erosion on my plots | 2 | Agronomic practices | 2 | Will be destroyed by animals |
| 3 | To reduce soil erosion on downstream plots | 3 | Agroforestry practices | 3 | Inconvenient for farm operation |
| 4 | To meet government interest | 4 | Soil and conservation practices | 4 | Scarcity of livestock feed |
| 5 | To conserve water | 5 | Other, specify? _________ | 5 | Suppress crop growth |
| 6 | To meet wood demand |  |  | 6 | Lack of knowledge |
| 7 | To produce feed for cattle |  |  | 7 | Scarcity of the necessary material |
| 8 | I don’t know |  |  | 8 | Other, specify ______________ |
| 9 | Other, specify ______________ |  |  |  |  |

**Section IV. Market participation and credit access**

**8. Access to output markets**

8.1. Did you sell any of the crop outputs you produced in 2017 (Ethiopian 2009/10 production year) [_______] **(Code-A)**

If the answer to question 8.1 is YES, fill the table, if NO skip to question 9

|  | 8.2 | 8.3 | 8.4 | 8.5 |
| --- | --- | --- | --- | --- |
| **No.** | **Crops sold** | | | |
|  | Crop type **(Code-Rc)** | Unit **(Code-Ra)** | Qty sold | Price per unit (Eth. Birr) |
| 1 |  |  |  |  |
| 2 |  |  |  |  |
| 3 |  |  |  |  |
| 4 |  |  |  |  |
| 5 |  |  |  |  |

**9. Access to Credits**

9.1. Did any member of your household member acquired loan in the last year 12 months? [_______] **(Code-A)**

If the answer to question 9.1 is YES, fill the table, if NO skip to question 10

| **No.** | | | 9.2 | 9.3 | | | 9.4 | |
| --- | --- | --- | --- | --- | --- | --- | --- | --- |
|  |  |  | **Loan type (Code-R)** | Amount you received **(in Eth. Birr)** | | | The main purpose of the loan **(Code-S)** | |
| **1** | | |  |  | | |  | |
| **2** | | |  |  | | |  | |
| **3** | | |  |  | | |  | |
| Loan type (Code-R) | | | | Loan purpose (Code-S) | | |  |  |
| 1 | Informal loan (relative, trader, etc.) | | | 1 | Purchase agricultural inputs | |  |  |
| 2 | Social organizations (*idir, ekub*, etc.) | | | 2 | Purchase livestock | |  |  |
| 3 | Microfinance institution | | | 3 | Expand/start microenterprise | |  |  |
| 4 | RuSACCO | | | 4 | Purchase farm tools | |  |  |
| 5 | Marketing cooperative | | | 5 | School expenditure | |  |  |
| 6 | Formal bank | | | 6 | Other consumption expenditure | |  |  |
| 7 | Other, specify _____________ | | | 7 | Other, specify _____________ | |  |  |

**10. Access to extension services**

10.1. Did you receive any extension advise /trainings/ about crop production and soil management in the past 12 months? [_______] (Code-A)

If the answer to question 10.1 is YES, fill the table below; If NO skip to question

| No. | | Source of extension advise **(Code-T)** | | 10.2 | | | 10.3 | 10.4 | | 10.5 |
| --- | --- | --- | --- | --- | --- | --- | --- | --- | --- | --- |
|  |  |  |  | What kind of extension advise did you get? **(Code-U)** | | | Was the advice timely? **(Code-A)** | Have you applied it?  **(Code-A)** | | Did you find the service useful? **(Code-A)** |
| 1 | |  | |  | | |  |  | |  |
| 2 | |  | |  | | |  |  | |  |
| 3 | |  | |  | | |  |  | |  |
| 4 | |  | |  | | |  |  | |  |
|  | | Source (Code-T) | | Topic of extension advise/training (Code-U) | | | |  |  |  |
| 1 | | Development agent (DA) | | 1 | Pest and disease | | |  |  |  |
| 2 | | Government offices | | 2 | Market and price | | |  |  |  |
| 3 | | Research center | | 3 | Good farm operation | | |  |  |  |
| 4 | | Peer groups | | 4 | Improved production technologies | | |  |  |  |
| 5 | | Farmers Training Center (FTC) | | 5 | Soil and water management | | |  |  |  |
| 6 | | Online group (facebook, WhatsApp, ..) | | 6 | Post-harvest technologies | | |  |  |  |
| 7 | | Media (TV, Radion…) | | 7 | Other, specify _____________ | | |  |  |  |
| 8 | | Other specify, _________ | |  |  | | |  |  |  |

**Section V: Social capital**

11.1. Did you belong to a farmer groups or organization in your community during the last 12 months? [______]**(Code-A)**

If Yes fill the table below. IF No skip to question 11.6

| No. | 11.2 | 11.3 | 11.4 | 11.5 |
| --- | --- | --- | --- | --- |
|  | Type of social group/organization  **(Code-V)** | Is there a Membership fee?  *1=Yes 0=No* | If yes in 14.3  How much in Eth. Birr | What is your main role in the Group  **(Code-W)** |
| 1 |  |  |  |  |
| 2 |  |  |  |  |
| 3 |  |  |  |  |

| Type of social group (Code-V) | | Role **(Code-W)** | |
| --- | --- | --- | --- |
| 1 | Women group | 1 | Committee member |
| 2 | Youth group | 2 | Ordinary member |
| 3 | RuSACCO | 3 | Other, specify _______ |
| 4 | Marketing Cooperatives/Association |  |  |
| 5 | Community resource management group |  |  |
| 6 | PA (Administrative/Militia, Cadre, etc.) |  |  |
| 7 | Other, specify_____________ |  |  |

**Section VI. Follow up**

If you don’t mind, could you share your phone number or any other phone number so that I can call you for any clarification on your responses? Phone number [_________________]

**Conclusion**

Ending time [ ]

Thank you very much for your time

Reference for Codes

| **Unit codes (Ra)** | | **Plant - (Code-Rc)** | | | |
| --- | --- | --- | --- | --- | --- |
| **Area – (Code-Ra1)** | | **Cereals – (Code-Rc1)** | | **Fruits – (Code-Rc4)** | |
| 1 | Gezem | 0 | Fallow | 36 | Bananas |
| 2 | Gemed | 1 | Teff | 37 | Mango |
| 3 | Timad | 2 | Barley (Gebis) | 38 | Orange |
| 4 | Hectare | 3 | Wheat (Durrah, Sinde) | 39 | Papaya |
| 5 | Medeb | 4 | Maize (Bekolo) | 40 | Lemmon |
| 6 | Square Meter | 5 | Sorghum (Mashila) | 41 | Other fruits |
| 7 | Sefera (40*25) | 6 | Corn (Zengada) | **Perennials tree – (Code-Rc5)** | |
| 8 | Guaro (20*25) | 7 | Oats (Aja) | 42 | Gesho |
| 9 | Kufaro | 8 | Sinar | 43 | Eucalyptus |
| 10 | Kedemia | 9 | Millet (Dagussa) | 44 | Bisana |
| 11 | Massa | 10 | Other cereals | 45 | Wanza |
| 12 | Ermija | **Pulses and oilseeds – (Code-Rc2)** | | 46 | Sesa |
| **Volume – (Code-Ra2)** | | 12 | Horse Beans (Bakela) | 47 | Girar |
| 1 | Kuna | 13 | Field Peas (Ater) | 48 | Fruit tree |
| 2 | Akmada | 14 | Chick Peas (Shimbra) | 49 | Susbania |
| 3 | Madaberia | 15 | Guaya (Vetch) | 50 | Others, specify |
| 4 | Dawla | 16 | Haricot Bean (Boloke) | **Grazing land – (Code-Rc6)** | |
| 5 | Silicha | 17 | Linseed (Telba) | 51 | Grassland |
| 6 | Sahin | 18 | Lentils (Mesir) | 52 | Feed trees |
| 7 | Kilograms | 19 | Sunflower (Suf) |  |  |
| 8 | Quintal | 20 | Nueg /Nigger seed/ |  |  |
| 9 | Chinet | 21 | Fenugreek (Abish) |  |  |
| 10 | Packets | 22 | Ginger (Zinjibel) |  |  |
| 11 | Tassa | 23 | Gibto |  |  |
| 12 | Liters | 24 | Others |  |  |
| 13 | Pieces | **Vegetables – (Code-Rc3)** | |  |  |
| 14 | Ensira | 25 | Potatoes |  |  |
| 15 | Kubaya | 26 | Onions (Key Shinkurt) |  |  |
| 16 | Birchiko | 27 | Spinach (Quosta) |  |  |
| 17 | Sini | 28 | Garlic (Nech Shinkurt) |  |  |
| 18 | Kerchat/Kemba | 29 | Sweet Potato |  |  |
| 19 | Number | 30 | Tomato |  |  |
| 20 | Dirib | 31 | Cabbage (Tikil Gomen) |  |  |
| 21 | Small Madaberia | 32 | Beet Root (Key Sir) |  |  |
| 22 | Big Madaberia | 33 | Carrot |  |  |
| 23 | Melekia/Lik | 34 | Lettuce (Selata) |  |  |
| 24 | Kolela | 35 | Local Cabbage (Gomen) |  |  |

**Codes related to carbon enhancing activities**

| **Soil and water conservation and management practices - (Code-Rf)** | | | | | |
| --- | --- | --- | --- | --- | --- |
| **A. Mechanical structures - (Code-Rf1)** | | **B. Conservation tillage- (Code-Rf2)** | | **D. Agroforestry practice - (Code-Rf4)** | |
| 1 | Soil/Stone bund | 9 | Contour tillage | 18 | Agroforestry trees |
| 2 | Terrace | 10 | Ridging and ridge tying | 19 | Grass strips |
| 3 | Fayna juu terrace | 11 | Minimum or zero tillage | 20 | Allay cropping (e.g. Susbania) |
| 4 | Check dam | 12 | Other, specify | 21 | Hedge |
| 5 | Diversion canal | **C. Agronomic practice - (Code-Rf3)** | | 22 | Other, specify |
| 6 | Interception ditches | 13 | Crop rotation |  |  |
| 7 | Gabion | 14 | Strip cropping |  |  |
| 8 | Other, specify | 15 | Mulching |  |  |
|  |  | 16 | Manuring |  |  |
|  |  | 17 | Other, specify |  |  |
|  |  |  |  |  |  |
